# Goat Milk Foodomics. Dietary Supplementation of Sunflower Oil and Rapeseed Oil Modify Milk Amino Acid and Organic Acid Profiles in Dairy Goats

**DOI:** 10.3389/fvets.2022.837229

**Published:** 2022-03-24

**Authors:** Einar Vargas-Bello-Pérez, Jaloliddin Khushvakov, Yongxin Ye, Nanna Camilla Pedersen, Hanne Helene Hansen, Lilia Ahrné, Bekzod Khakimov

**Affiliations:** ^1^Department of Veterinary and Animal Sciences, Faculty of Health and Medical Sciences, University of Copenhagen, Frederiksberg, Denmark; ^2^Department of Food Science, Faculty of Science, University of Copenhagen, Frederiksberg, Denmark; ^3^Institute of Chemistry and Biotechnology, School of Life Sciences and Facility Management, Zurich University of Applied Sciences, Wädenswil, Switzerland

**Keywords:** goats, unsaturated fatty acids, vegetable oils, sunflower oil, rapeseed oil, foodome

## Abstract

The dietary supplementation of vegetable oils is known to improve the dietary energy density as well as milk fatty acid profile; however, the impacts on the milk foodome is largely unknown. This study investigated the effect of two different sources of unsaturated fatty acids, rapeseed oil and sunflower oil, as a feeding supplement on the milk foodome from dairy goats. Nine Danish Landrace goats at 42 ± 5 days in milk were allocated to three treatment groups for 42 days with three animals per group. A control group received a basal diet made of forage and concentrate at an 85:15 ratio. On top of the basal diet, the second and third groups received rapeseed oil or sunflower oil supplements at 4% of dry matter, respectively. Goat milk was sampled on days 14, 21, and 42. The milk foodome was measured using gas chromatography–mass spectrometry and proton nuclear magnetic resonance spectroscopy. The milk levels of 2-hydroxyisovaleric acid, oxaloacetic acid, and taurine were higher in the milk from goats fed with sunflower oil compared to the control group. More glucose-1-phosphate was found in the milk from goats fed with rapeseed oil compared to the control group. Amino acids, valine and tyrosine, and 2-hydroxyisovaleric acid and oxaloacetic acid were higher in the sunflower group compared to the rapeseed group, while the milk from the rapeseed-fed goats had greater levels of ethanol and 2-oxoglutaric acid compared to the sunflower group. Thus, results show that foodomics is suitable for studying how milk chemistry changes as a function of feeding regime.

## Introduction

The fat content of goat milk and its chemical composition have been extensively modulated by nutritional and genetic factors (1, 2). The most practical way to modulate milk fatty acids (FAs) toward a healthier profile for human consumption is by the supplementation of animal feed with vegetable oils (3–5). The chemical changes in milk FA profile depend on the FA profile of vegetable oil supplement, such as length of FA chain, degree of unsaturation, and orientation of FA double bonds as shown in an *in vitro* study performed on goat mammary gland cells (6).

When ruminants are fed with unsaturated FA sources, the milk FA profiles are changed because of the hydrogenation and isomerization of dietary unsaturated FA in the rumen (7). In goats, the dietary supplementation of rapeseed oil (8) and sunflower oil decrease the milk contents of saturated FAs (9, 10). However, the possible effects of lipid supplementation on goat milk foodome are unknown.

To the best of authors' knowledge, this is the first foodomics study performed on goat milk using the two complementary analytical techniques: derivatization-based untargeted gas chromatography–mass spectrometry (GC-MS) and proton (^1^H) nuclear magnetic resonance (NMR) spectroscopy. Foodomics is a scientific discipline investigating food molecular composition and nutritional properties as a function of food production processes and the impact of food on human health and wellbeing (11, 12). Similar to metabolomics (13), which studies the metabolic response (qualitative and quantitative changes in metabolites) of biological systems to an external stimuli (14, 15), foodomics studies the changes in the chemical composition (*foodome*) of food systems as a function of external stimuli and seeks an impact of these changes on the nutritional value of food and subsequently on human health. The application of unbiased foodomics approaches is promising for screening milk chemical composition as a function of the feeding regime (16, 17), identifying disease biomarkers (18, 19), and assessing the overall milk quality (20) or for predicting the technological properties of milk in the dairy industry (21, 22). A recent study showed that increased milk butanoic and hexanoic acid levels are related to methane production (23).

While several studies have been conducted to investigate milk FA profile changes as a function of feeding regime, not many studies looked into the nutritional–chemical “barcode” (foodome) of milk. This study explored the goat milk foodome as a function of dietary supplementation with vegetable oils including rapeseed oil or sunflower oil. This study demonstrates changes in the goat milk foodome, determined using GC-MS and NMR, as a function of dietary supplementation with two different sources of unsaturated FAs, rapeseed oil and sunflower oil, both at the concentration of 4% dry matter content.

## Materials and Methods

### Animals and Diets

The present study was conducted in compliance with the Danish Ministry of Justice Law No. 474 (May 15, 2014) concerning animal experimentation and the care of experimental animals. The study was performed at a goat farm located in Tureby, Denmark (55°19'38.9“N, 12°06'36.1”E). Nine Danish Landrace goats at 42 ± 5 days in milk at the beginning of the study were allocated to three treatment groups. At the onset of the study, the average body condition scores for the 3 groups were 3.2 ± 0.2, 3.0 ± 0.1, and 2.8 ± 0.2 (scored on a 5-point scale), while the body weights were 45 ± 4, 47 ± 5, and 42 ± 5 kg. The goats were housed with their kids in a stall (12 × 38 m) with continuous access to water. For 42 days, all animals received the same basal diet consisting of (% of dry matter) 21 lucerne + clover hay, 23 clover haylage, 42 lucerne + grass hay, and 14% straw. The basal diet was offered daily at 07:00 and a concentrate (300 g/d/animal) was supplied during milking at 10:00. The concentrate given to the control animals consisted (% of dry matter) of 93% of a grain mix (40 rolled barley, 30 rolled oats, 10 rolled wheat, and 20% rolled peas), 6 molasses, and 1% premix of vitamins and minerals. The dietary treatments contained an 85:15 forage-to-concentrate ratio. The diets were formulated to meet the nutrient requirements of a mature dairy doe with twin kids and a body weight between 40 and 50 kg according to the NRC (2007). The oil-supplemented concentrates consisted of (% of dry matter) 89 grain mix, 6 molasses, 1 premix of vitamins and minerals, and 4% of either sunflower oil or rapeseed oil. The oils were not rumen protected and were mixed manually into the daily concentrate of each goat. The chemical composition and FA profile from diets are shown in Table 1.

**Table 1:** Diet and treatment composition.

	**Diets**		
	**Control**	**Rapeseed oil**	**Sunflower oil**
**Chemical composition, % DM**			
Dry matter	82.5	83.2	82.8
Ether extract	2.39	6.92	6.93
Crude protein	1.40	1.38	1.35
Ash	3.18	3.18	3.40
Neutral detergent fiber	55.3	55.3	55.3
Acid detergent fiber	32.3	32.3	32.3
Lignin	4.8	4.9	4.8
**Fatty acids, g/100 g**			
C16:0	0.6	5.4	4.1
C18:0	1.0	3.5	0.7
C18:1	18.0	24.5	54.0
C18:2	45.6	56.1	24.7
C18:3	32.6	9.5	15.8
>C20	2.2	0.9	0.8

### Milk Production and Composition

The milk yield was recorded at 07:00 on days 14, 21, and 42 prior to a 12 h separation of does and their kids. For statistical analysis, these sampling dates were considered as experimental periods (0–14, 14–21, and 21–42 days). For the milk composition determination, the individual samples (50 ml) within treatment were pooled for each collection date and effect of treatment and were analyzed for fat, protein, lactose, casein, total solids, citric acid, solids-not-fat, urea, free FA, acidity, and density. These analyses were done in duplicate by Fourier transform infrared spectroscopy using a MilkoScan™ FT2 (Foss Analytical A/S, Hillerød, Denmark). For statistical analysis, the data were analyzed using a model that included treatment as a fixed effect and animal as a random effect in a randomized block design. Individual milk samples (200 ml) were taken at 10:00 on days 14, 21, and 42. All raw milk samples were stored at −4°C for further analysis. Individual milk samples were then analyzed using GC-MS and ^1^H NMR spectroscopy.

### Chemicals and Reagents

Analytical scale methanol (99.9%), dichloromethane (99.9%), sorbitol (98%), C10–C40 all-even alkane mixture (for GC-MS analysis), trimethylsilyl cyanide (TMSCN, 99.8%), monobasic potassium phosphate (KH_2_PO_4_, 99.0%), dibasic potassium phosphate (K_2_HPO_4_, 98.0%), deuterium oxide (D_2_O, 99.9 atom % D), sodium salt of 3-(Trimethylsilyl) propionic-2,2,3,3-d4 acid (TSP, 98 atom % D), and sodium azide (NaN_3_, 99.5%) (for NMR analysis) were purchased from Sigma-Aldrich (Søborg, Denmark). Water used throughout the study was purified using a Millipore lab water system (Merck KGaA, Darmstadt, Germany) equipped with a 0.22 μm filter membrane.

### Gas Chromatography-Mass Spectrometry (GC-MS)

Frozen milk samples were thawed at room temperature and vigorously vortexed until homogenization, and 1 ml of sample was placed into 15 ml falcon tubes. After sonication for 15 min, 200 μl of milk was transferred into 2.0 ml Eppendorf tubes, followed by addition of 300 μl of 80% methanol [containing 10 ppm internal standard (IS), sorbitol] and 100 μl of dichloromethane. This was followed by 10 min of vigorous vortex mixing at room temperature, and the centrifugation at 13,572 *g* for 10 min at 4°C. Then 100 μl of the upper aqueous layer was transferred into a 200-μl glass insert and dried overnight using ScanVac (Labogene, Lynge, Denmark) at 40°C and 1,000 rpm. Immediately after drying, the glass inserts were sealed with airtight magnetic lids into the GC-MS vials, stored at 4°C, and analyzed by GC-MS within 24 h. The GC-MS analysis was performed as previously described (24). Briefly, the dried milk extracts were trimethylsilylated using the derivatization reagent TMSCN (25); 40 μl of TMSCN was added, followed by agitation at 750 rpm for an hour at 60°C. Samples were randomized prior to GC-MS, and derivatization and injection were automated using a Dual-Rail MultiPurpose Sampler (Gerstel, Mülheim an der Ruhr, Germany).

The GC-MS consisted of an Agilent 7890B GC (Agilent Technologies, Santa Clara, CA, United States) coupled with an HT Pegasus time-of-flight mass spectrometer (LECO Corporation, Saint Joseph, MO, United States). The GC separation was performed on a Zebron ZB 5% phenyl 95% dimethylpolysiloxane column (30 m × 250 × 0.25 μm) with a 5 m inactive guard column (Restek Corporation, Bellefonte, PA, United States). Hydrogen was used as a carrier gas at the constant column flow rate of 1.0 ml/min. The initial temperature of the GC oven was 40°C, which was held for 2 min, and heated to 220°C at a rate of 10°C/min, followed by 55°C/min to reach a final temperature of 320°C at which the oven was kept for 6 min. The mass spectra were recorded in the range of 45–600 *m/z* with a data acquisition rate of 10 spectra·s^−1^. The MS detector and ion source were switched off during the first 6 min of solvent delay time. The transfer line and ion source temperature were set to 280 and 250°C, respectively. The GC-MS data were processed using the freeware program PARADISe (26). Metabolites were identified using the National Institute of Standards and Technology version 11 (NIST11) library either at level 2, electron impact–mass spectrum (EI-MS) match of ≥800 and retention index (RI) match of ± 30, or at level 3 when peaks were tentatively assigned to metabolite classes based on their spectral similarities (EI-MS match ≥650) (27).

### Proton (^1^H) Nuclear Magnetic Resonance (NMR) Spectroscopy

The frozen milk samples were thawed at room temperature and vigorously vortexed until they were homogenized, and 1.8 ml of milk samples were centrifuged (13,572 *g* for 30 min at room temperature). Then, 600 μl of aliquot from the clear solution was mixed with 135 μl of the phosphate buffer in deuterium oxide (D_2_O) and transferred into NMR SampleJet tubes (L = 103.5 mm and O.D. = 5.0 mm), kept at 5°C, and analyzed within 24 h. The phosphate buffer was prepared as follows: 1.5 M solutions of monobasic potassium phosphate (KH_2_PO_4_) and dibasic potassium phosphate (K_2_HPO_4_) were prepared in D_2_O and mixed in a 1:4 (vol:vol) ratio, respectively. Then, the sodium salt of 3-(trimethylsilyl) propionic-2,2,3,3-d4 acid (TSP) and sodium azide (NaN_3_) were added to the phosphate buffer at the concentrations of 1.0 and 0.13 mg/ml, respectively.

One-dimensional (1D) ^1^H NMR spectra were acquired at the Department of Food Science (University of Copenhagen) as previously described (28). A Bruker Avance III 600 MHz NMR spectrometer equipped with a 5 mm broadband inverse RT (BBI) probe, automated tuning and matching accessory (ATMA), and cooling unit BCU-05, and an automated sample changer (SampleJet; Bruker BioSpin, Rheinstetten, Germany) with sample cooling (5°C) and preheating stations (25°C). Data acquisition and processing were carried out using the software TOPSPIN 3.6.2 PL5 (Bruker BioSpin, Rheinstetten, Germany). The sample was preheated at 25°C for 60 s in SmapleJet and kept inside the NMR probe head for 3 min in order to reach temperature equilibrium at 27 ± 0.1°C. Then, automated tuning and matching, automated locking, and automated shimming (TOPSHIM routine) were performed. The 1D ^1^H NMR spectra were acquired using the standard pulse sequence with water suppression *noesygppr1d* from the Bruker pulse program library.

Sixty-four scans were acquired, after four dummy scans, and the generated free induction decays (FIDs) were collected into 98 k data points using a spectral width of 30 ppm. The acquisition time, relaxation delay, and mixing time were set to 2.72, 4.0, and 0.01 s, respectively. The receiver gain was set to 128 for all samples. The automation program used to control sample measurements included, together with the acquisition routines for locking, automated tuning and matching, and shimming, the 90° hard pulse calibration and optimized presaturation power for each sample, as well as automated data processing including the Fourier transformation of FID, with a line-broadening of 0.3 Hz, automated phasing, and baseline correction. 1D ^1^H NMR spectra were imported into the SigMa software (29), referenced to the TSP singlet at 0 ppm, and the spectral intervals with a significant shift were aligned using *icoshift* (30) implemented in SigMa. The assignments of signals forming the NMR spectra were carried out using the Milk Composition Database (31) and the data from the previous studies (32–34).

### Data Analysis

The univariate analysis and one-way ANOVA with Benjamini–Hochberg's multiple test correction approach (35) using the false discovery rate of 10% were applied to investigate the possible effect of the dietary treatment and the period on the goat milk foodome data measured by GC-MS and ^1^H NMR. Bartlett's test of sphericity was applied to examine the hypothesis that the variables were uncorrelated.

And the Kaiser–Meyer–Olkin index to measure the sampling adequacy with factorial analysis. A hierarchical clustering analysis was also performed, and a discriminant analysis verified the extent to which the samples were correctly assigned to the clusters identified in the previous analysis. Principal component analysis (PCA) (36) and ANOVA simultaneous component analysis (ASCA) (37) with the permutation test (*n* = 2,000) was performed as previously described (11, 15). Prior to PCA and ASCA, the foodomics datasets were mean centered and scaled to a unit variance (autoscaled). All data analyses were performed in MATLAB version R2016b (The MathWorks Inc., Natick, MA, United States) using the customized scripts written by the authors.

## Results

### Milk Production and Composition

The milk yield was not affected by the dietary supplementation of sunflower oil and rapeseed oil (1.06 ± 0.16 kg/d; *P* = 0.056). The milk concentrations of fat (2.18 ± 0.17/100 g; *P* = 0.356), protein (2.61 ± 0.16/100 g; *P* = 0.155), lactose (4.49 ± 0.25/100 g; *P* = 0.106), casein (2.05 ± 0.19/100 g; *P* = 0.171), total solids (10.0 ± 0.45/100 g; *P* = 0.121), citric acid (0.11 ± 0.01/100 g; *P* = 0.180), solids-not-fat (7.98 ± 0.39/100 g; *P* = 0.091), urea (30.2 mg/100 ml; *P* = 0.363), free FAs (0.78 ± 0.07 mEq/L; *P* = 0.759), acidity (10.6 ± 0.74 °Th; *P* = 0.346), and density (1,026 ± 1.56 kg/L; *P* = 0.085) were similar between the treatments.

### Milk Foodome

The untargeted GC-MS analysis of goat milk samples allowed the detection of 97 peaks, and 38 out of them were identified at level 2 with a close match of mass spectra and retention indices to the National Institute of Standards and Technology (NIST) library, and 24 compounds were tentatively assigned with a low degree of precision and belong annotated to level 3 (metabolite class level only) (Supplementary Table S1). Level 2 identified metabolites included organic acids, amino acids, and sugars, while the majority of level 3 metabolites were tentatively annotated as sugars and sugar alcohols. The thirty-five peaks detected from the GC-MS analysis remained unknown. The ^1^H NMR spectra acquired on goat milk samples revealed 116 resonances, and 68 of these represented one or more signals streaming from 48 milk metabolites (Supplementary Table S2). These included different classes of molecules such as short-chain FAs, amino acids, organic acids, sugars, amines, choline, purine, and pyrimidine derivatives. In contrast to GC-MS, NMR also allowed the quantification of lactose, which was not detected by GC-MS since the method was developed to avoid an overloading signal of this major milk sugar. ^1^H NMR spectra was largely dominated by the signals from lactose, urea, glucose, galactose, and citric acids, in the descending order (data not shown). In addition, ^1^H NMR spectra revealed 21 intervals that were quantified as bins (by summing data points) that represent the overlapped signals of more than one metabolite. These complex regions contained chemical medium-chain FAs, amino acids, organic acids, sugars, and their derivatives, quaternary ammonium derivatives. The twenty-seven signals detected from the ^1^H NMR spectra remained unknown.

### Effect of Dietary Treatment on Goat Milk Foodome

**Figure 1:**
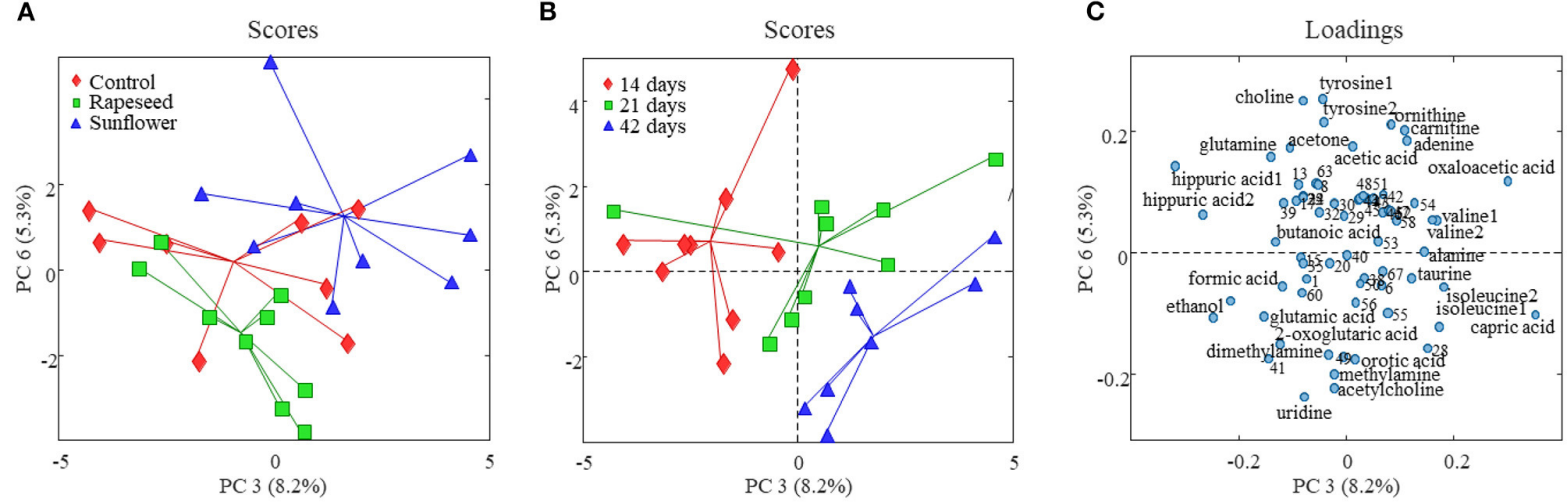
Score and loading plots of the PCA model developed on goat milk NMR foodomics data. **(A)** PC3 vs. PC6 score plot and milk samples are color coded according to the dietary treatment, and in panel **(B)**, milk samples are colored according to the experimental period. **(C)** PC3 vs. PC6 loading plot from the corresponding PCA model (variables are numbered as in Supplementary Table S2).

The effect of dietary treatment on the milk foodome measured using GC-MS and NMR data was explored using PCA (Supplementary Figures S1, S2). The first two principal components (PCs) of the PCA model developed using the NMR data explained just above 45% variation present in the data. However, the score plot (PC1 vs. PC2) showed no trend of separation of milk samples according to the dietary treatment. Higher PCs, PC3 (explained 8% variation) vs. PC6 (5%), of the same PCA model captured the variation related to the dietary treatment and showed a partial separation of milk samples according to the oil supplementation (Figure 1A). PC6 separated the three feeding regimes, where the control treatment was in the middle between the rapeseed (lower PC6 scores) and sunflower (higher PC6 scores) treatments. PC3 largely separated sunflower from the other two treatments. Corresponding loadings of the same PCA model (Figure 1C) suggests that goats fed with rapeseed oil represented a milk foodome relatively higher in concentrations of ethanol, glutamic acid, 2-oxoglutaric acid, pyrimidine derivatives including uridine and orotic acid, amines such as methylamine and dimethylamine, and acetylcholine. While the milk samples from goats fed with sunflower oil appeared on top-right side of the score plot due to relatively higher concentrations of adenine, quaternary ammonium derivatives (choline and carnitine, amino acids (tyrosine and ornithine), and organic acids (oxaloacetic acid and acetic acid).

**Figure 2:**
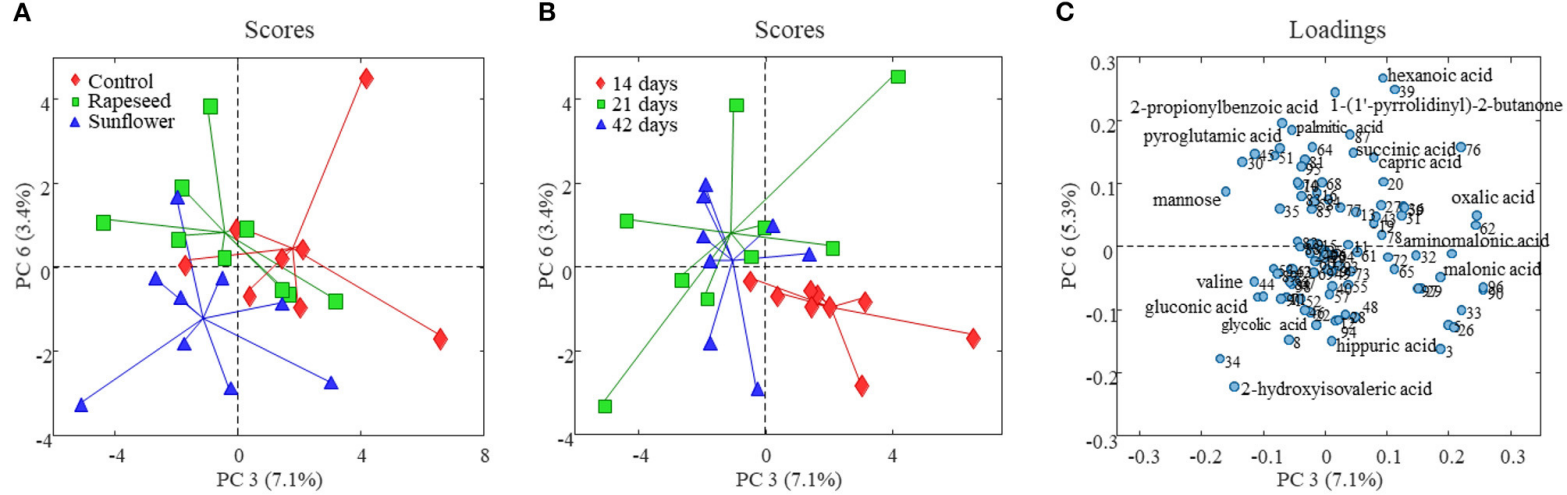
Score and loading plots of the PCA model developed on goat milk GC-MS foodomics data. **(A)** PC3 vs. PC6 score plot and milk samples are color coded according to the dietary treatment, and in panel **(B)**, milk samples are colored according to the experimental period. **(C)** PC3 vs. PC6 loading plot from the corresponding PCA model (variables are numbered as in Supplementary Table S1).

The PCA of the GC-MS data revealed no systematic variation related to dietary treatments on the first two components that explained almost 65% variation (Supplementary Figure S2A). Similar to the NMR data, the PCA model developed on the GC-MS data captured a variation explaining dietary treatment on PC3 (7%) vs. PC6 (3%) (Figure 2A). PC3 partially distinguished milk samples of the control group from sunflower and rapeseed oil groups, while PC6 mostly separated sunflower from the other two treatments. The corresponding loading plots (Figure 2C) indicated that the milk samples from the sunflower group were characterized by having relatively higher concentrations of 2-hydroxyisovaleric acid, valine, glycolic acid, and gluconic acid and lower levels of hexanoic acid, oxalic acid, and succinic acid compared to the two other groups. The milk from rapeseed oil-fed goats was characterized with relatively higher levels of pyroglutamic acid, 2-propionylbenzoic acid, palmitic acid, and mannose. The milk samples from the control group were characterized by greater levels of oxalic acid, hexanoic acid, and capric acid.

In order to investigate an impact of dietary treatments on individual metabolite levels, a one-way ANOVA was performed. A total of 8 metabolites, 7 from the NMR and one from GC-MS (2-hydroxyisovaleric acid), were different between the three dietary treatments (Tables 2, 3). These metabolites were also a part of the metabolic patterns responsible for separating milk samples in the PCA score space according to the treatment groups (Figures 1C, 2C). The seven metabolites detected by NMR included valine, ethanol, oxaloacetic acid, 2-oxoglutaric acid, taurine, tyrosine, and glucose-1-phosphate. The levels of valine, tyrosine, taurine, oxaloacetic acid, and 2-hydroxyisovaleric acid were higher in milk samples from the sunflower oil group compared to the rapeseed oil group (*P* < 0.05). The milk levels of ethanol and 2-oxoglutaric acid were higher in the rapeseed group compared to the sunflower oil group (*P* < 0.05). Glucose-1-phosphate was also highest in the rapeseed group but was only significantly different from the control group (*P* = 0.012).

**Table 2:** Effect of rapeseed oil (RS) and sunflower oil (SF) supplementation on milk metabolites from goats.

**# Metabolite**	**Metabolite**	**RS/CN**	**SF/CN**	**Variation (%)**	**SEM**	***P***
19	Oxaloacetic acid	0.67	3.62^b^^,^^c^	37.55	2.90E + 05	0.004
27	2-Oxoglutaric acid	1.23	0.87^c^	34.73	0.49E + 05	0.007
5	Ethanol	2.09	0.46^c^	32.19	0.50E + 05	0.011
55	Glucose-1-phosphate	1.32^a^	1.12	32.00	0.14E + 05	0.012
35, 36, 40	Taurine	1.50	1.84^b^	29.51	0.56E + 05	0.018
61, 62	Tyrosine	0.41	1.25^c^	27.81	0.25E + 05	0.024
2, 4	Valine	0.62	1.49^c^	22.22	1.01E + 05	0.056
17	2-Hydroxyisovaleric acid	0.65	4.31^b^^,^^c^	29.92	1.05E + 05	0.017

*#Metabolite corresponds to the NMR variable numbers in Supplementary Table S2. RS/CN is the ratio of the mean abundance of a metabolite in milk from rapeseed oil-fed goats and the control group as follows: mean (rapeseed,i)/mean (control,i), where i is a metabolite. The ratios of metabolites between sunflower oil and the control (SF/CN) group were calculated in the same way. Variation % and P-value were obtained from a one-way ANOVA performed for the treatment effect*.

a *Indicates P < 0.05 in multiple test comparisons between rapeseed oil and the control group*.

b *Indicates P < 0.05 in multiple test comparisons between sunflower oil and the control group*.

c *Indicates P < 0.05 in multiple test comparisons of rapeseed oil and sunflower oil*.

**Table 3:** Effect of collection date (14, 21, and 42 days) on milk metabolites from goats.

**# Metabolite**	**Metabolite**	**21 day/14 day**	**42 day/14 day**	**Variation (%)**	**SEM**	***P***
23	Methylamine	0.36^a^	1.04^c^	54.11	0.19E + 05	0.0001
64, 65, 66	Hippuric acid	0.65^a^	0.48^b^	51.67	0.38E + 05	0.0002
16	Acetone	0.31^a^	0.48^b^	49.40	0.32E + 05	0.0004
7	Capric acid	2.76^a^	3.08^b^	48.86	10E + 05	0.0004
21	Glutamine	0.55^a^	0.58^b^	48.40	1.4E + 05	0.0005
28	Creatine	1.58^a^	1.92^b^	43.87	4.2E + 05	0.0013
2, 4	Valine	2.82^a^	2.67^b^	36.75	1.3E + 05	0.0052
26	Dimethylamine	0.21^a^	1.03^c^	34.61	0.90E + 05	0.0076
31	Choline	0.70	0.48^b^	34.35	0.98E + 05	0.0079
12, 13	Ornithine	0.54^a^	0.63^b^	34.17	0.17E + 05	0.0082
10	Alanine	1.37	1.59^b^	32.52	0.80E + 05	0.0109
19	Oxaloacetic acid	6.13	8.18^b^	24.74	2.4E + 05	0.0380
3, 9	Isoleucine	1.33	1.76^b^	23.64	0.19E + 05	0.0450
69	Formic acid	0.31^a^	0.48	23.21	0.58E + 05	0.0480
92	Palmitic acid	1.75	5.63^b^^,^^c^	30.71	0.08E + 05	0.0147

*#Metabolite corresponds to NMR variable numbers in Supplementary Table S2. 21 day/14 day is a fold change calculated between the mean abundance of a metabolite in all 21-day milk samples and 14-day milk samples as follows: mean (21 day, i)/mean (14day, i), where i is a metabolite. The fold changes of metabolites between 42 days and 1 day (42 days/1day) were calculated in the same way. Variation % and P-value were obtained from a one-way ANOVA performed for the period effect*.

a *Indicates P < 0.05 in multiple test comparisons of 21- and 14-day milk*.

b *Indicates P < 0.05 in multiple test comparisons of 42- and 14-day milk*.

c *Indicates P < 0.05 in multiple test comparisons of 21- and 42-day milk*.

### Effect of Experimental Period on Goat Milk Foodome

The effect of the period was more pronounced on the milk foodome than dietary treatments. The PCA performed on NMR and GC-MS data showed a partial separation of milk samples according to the date of collection at 14, 21, and 42 days (Supplementary Figures S1B, S2B). Similar to the dietary treatment, the PC3 (8%) vs. PC6 (5%) score plot of the model generated on NMR data showed better separation of milk according to the experimental date (Figure 1B). Along the PC3, all three periods are clustered consecutively, while PC6 mainly separated 42-day milk samples from the other two. Acetone, butanoic acid, glutamine, tyrosine, and hippuric acid were the main metabolites characterizing (present at higher amounts) 14-day milk, whereas isoleucine, caprolic acid, creatine, choline, taurine, and glucose-1-phosphate were higher in 42-day milk compared to the other two experimental periods (Figure 1C). A partial separation of the three experimental periods was also observed from the PCA analysis of the GC-MS data (Figure 2B). The score plot, PC3 (7%) vs. PC6 (3%), depicted only the separation of 14-day milk samples from 21- and 42-day milk samples. The milk metabolites responsible for this separation included malonic acid, serine, and aminomalonic acid, which was greater at 14 days, and capric acid, 1-(1'-pyrrolidinyl)-2-butanone, 2-hydroxyisovaleric acid, pyroglutamic acid, 2-propionylbenzoic acid, α-D-mannopyranose, scyllo-inositol, and palmitic acid that were greater in the milk from 21 and 42 days.

Fourteen metabolites detected by NMR and palmitic acid detected by GC-MS were differed significantly by period (Tables 2, 3). The two amines in milk samples, methylamine and dimethylamine, declined in relative concentration from 14 to 21 days (up to 70%), followed by a recovery in the 42 days of milk period. A similar trend of fluctuation was observed also for glutamine, ornithine, acetone, and formic acid, but with less recovery by the 42nd day, whereas the relative mean levels of the milk FAs, capric acid and palmitic acid, and the amino acid valine increased 2–5-fold throughout the duration of the experiment. Similarly, but to a lesser extent, the milk levels of non-protein nitrogen-containing compounds including creatine, isoleucine, and alanine increased during the experimental period. Surprisingly, the relative mean level of oxaloacetic acid in milk increased six and eight times from days 14 to 21 and days 14 to 42, respectively. In contrast, the relative mean levels of choline, hippuric acid, and glutamine in milk decreased up to 50% during the experimental periods.

## Discussion

The lack of differences in milk production and composition was expected since the amount of dietary lipid supplements was low and did not compromise animal performance. Our findings agree with the previous studies in mountain-grazing goats fed with rapeseed oil or hydrogenated palm oil at 8% DM. There were no changes in the milk yield throughout the experimental period (38). Similarly, dairy goats fed diets enriched with whole linseed oil (0.81% DM) or sunflower oil (1.84% DM) had no impact on milk yield or on the content of fat, protein, or total solids (9). Compared with cows, goats are more resilient to lipid supplements as they have less sensitivity to the antilipogenic effects of some *trans* FA isomers during mammary lipogenesis (39), and this might be an explanation for the lack of differences between treatments over milk fat. On the other hand, the period effect (14, 21, and 42 days) was more pronounced on the milk foodome than the dietary treatments. This is partly due to the relatively low amount of lipid used (4% DM), which is also reflected in the lack of changes in milk composition. Another explanation is because of the inherent physiological changes due to lactation. This has been shown in humans' milk metabolite profiles, from colostrum to milk from the first 2 weeks postpartum (40) and in bovines, from colostrum to the first milk after calving (41), but until now, no data are available from goats. This shows that milk metabolites undergo significant changes over short periods of lactation, and this was also reflected during the experimental periods of the present study.

An increased level of ethanol in milk from goats fed with rapeseed oil could be related to a decreased pH in the rumen, which is related to a high cereal grain-based diet (42). In this context, ethanol facilitates the translocation of endotoxin through rumen and colon walls, leading to endotoxin-related disorders (42). Another increase of ethanol in milk was also found to be related to roughage feeding, where a higher ethanol concentration was observed in milk samples from cows fed with maize silage- based diet compared to grass-silage based diets (18 and 1.87 μg/kg) (43). Thus, feeding rapeseed oil to goats seems to compromise rumen health as indirectly indicated by the increase in milk ethanol. To corroborate these findings and investigate the mechanisms of increased ethanol, further studies should be performed including rumen metabolite profiling.

The rapeseed oil-fed goats had significantly increased levels of glucose-1-phosphate, compared to the (up to 30%) control diet and significantly higher levels of 2-oxoglutaric acid, compared to the sunflower group. The increase of glucose-1-phosphate in rapeseed oil-fed group is a striking finding since there is an evidence that glucose availability is important for the expression of genes involved in milk fat synthesis and lactose and glucose metabolism in bovine mammary epithelial cells (44). An increased level of glucose-1-phosphate indicates that, compared to sunflower oil, rapeseed could be a promising dietary lipid source since it improves the technological properties of milk for cheese manufacturing (increased total solids). However, taking in consideration that ethanol was increased, further studies should make a trade-off between the dietary inclusion of rapeseed oil and the duration of such feeding regime as it seems to compromise the animal's health. The milk from goats fed with rapeseed oil had more 2-oxoglutaric acid, which is an intermediate in the tricarboxylic acid cycle and is the starting point in the formation of glutamate and the other members of the glutamate family (45). In high-yielding cows, glutamine has been proposed to be a limiting factor for milk protein synthesis (46). Thus, the increased 2-oxoglutaric acid warrants further attention as it may compromise milk protein synthesis or be a sign of decreased glutamate synthesis.

In addition to these significantly different milk metabolites, the metabolite pattern responsible for the separation of the rapeseed group on the PCA score space included glutamic acid; pyrimidine- derivatives including uridine and orotic acid; and amine and quaternary ammonium derivatives such as acetylcholine, methylamine, and dimethylamine. The fact that rapeseed oil increased the presence of amine and quaternary ammonium derivatives may be related to favorable conditions for the growth and activity of the pathogenic bacteria in the rumen, which has been related to the high dietary grain supply in ruminants (42). Further studies should consider analyzing the rumen metabolome and milk foodome in order to confirm our results about amine presence. However, in this study, the animals did not show signs of either subclinical or clinical acidosis or any other type of digestive disorder.

The milk metabolites that increased with the sunflower oil diet are related to protein metabolism. For example, oxaloacetic acid, an intermediate product in the tricarboxylic acid cycle, plays important roles in regulating mitochondrial function, including gluconeogenesis, urea recycling, and amino acid syntheses (47). Taurine is an end-product from the metabolism of methionine and cysteine (48). Taurine in milk also plays an important role in the growth and development of newborns (49). Taurine is the most preponderant free amino acid in goat milk and considered to be an important milk nutrient for very young babies or preterm infants (50). Human and goat milk have a similar content of this amino acid, which is one of the reasons why goat milk is a good alternative to human milk (50). Tyrosine, which also increased in the milk from sunflower oil-fed goats, has been proposed as an indicator for energy balance in early lactating cows where the cows in negative energy balance have lower levels of tyrosine in milk (51). Given the increased tyrosine in this study, it is possible to hypothesize that sunflower oil improved energy balance in dairy goats. Meanwhile, valine, a branched chain amino acid, is involved in the biosynthesis of branched-chain FA (21) and thus contributes to milk flavor (52). A particular “goaty” flavor of goat milk is mainly due to straight-chain free FA, mainly C6:0 to C9:0 (38), and branched-chain free FA, C9:0 and C10:0 (53). The milk foodome from goats fed with sunflower oil suggests that this lipid source could be used to increase specific sensory characteristics such as flavor and at the same time improve the presence of nitrogen and lipids that are needed for dairy product manufacturing.

Among all metabolites, 2-hydroxyisovaleric acid showed the greatest increase in the milk from sunflower oil-fed goats compared to the other two groups (up to 4-fold). Also, 2-hydroxyisovaleric acid showed the same tendency as tyrosine and valine, and this metabolite during fermentation processes is related to the production volatile acids, which in turn promotes the formation of rumen acetic and butanoic acids that are needed for milk fat synthesis (7). On the other hand, the increase in acetic acid, butanoic acid, and lactic acid in milk is related to mammary gland infections (32, 54); however, in this study, animals were monitored for mastitis and no clinical cases were found. From a food perspective, butanoic acid is one key compound associated with aroma profile of dairy products and it is responsible for strong and pungent odors (55).

Rapeseed or sunflower oils have been studied before as feeding supplements for goats. However, for the first time, the effects of those dietary oils on milk have been analyzed using an untargeted foodomics approach. It was also shown that nitrogenous compounds can be increased in the milk from goats fed with sunflower oil. This suggests that sunflower oil supplementation could be used as a means for improving the milk quality for dairy product manufacturing. Dietary fat reduces milk protein contents in cows as it affects rumen microbial protein yields and the availability of amino acids for gluconeogenesis (6). In a meta-analysis focused on the data from the cheeses from the sheep fed with different sources of dietary unsaturated FA, the milk protein contents were not affected (56). In the current study, the increases in nitrogenous compounds may be explained by the fact that the amount of dietary lipids was not enough to negatively alter protein metabolism at the rumen level. It is worth mentioning that this exploratory research was conceived as a nutritional intervention that was focused on the application of foodomics approach for studying goat milk. As such, the number of animals used for the study was limited. The goat was chosen as a model due to its lower sensitivity to dietary lipids. Further studies should consider correlating rumen metabolite profiles with the milk foodome as well as quantifying the chemical and sensory characteristics of the resulting dairy products. Then, the findings from this study on animal health and food quality will be corroborated.

## Conclusion

The goat milk foodome, for the first time, has been characterized using two complementary analytical techniques, derivatization-based untargeted GC-MS and ^1^H NMR spectroscopy. The study showed minor effects on the milk foodome when goats are supplemented with vegetable oils that were mainly manifested in energy metabolism and protein synthesis. Feeding goats with sunflower oil at 4% DM dietary inclusion can increase the relative concentrations of milk amino acids and organic acids, while rapeseed oil increased aliphatic alcohols, including ethanol and organic acids. Additionally, our results showed that the unbiased ^1^H NMR approach resulted in more milk metabolite variations related to the oil supplements compared to GC-MS, which is a more suitable approach to quantify volatile and semi-volatile metabolites.

## Data Availability Statement

The original contributions presented in the study are included in the article/Supplementary Material, further inquiries can be directed to the corresponding author/s.

## Ethics Statement

The present study was conducted in compliance with the Danish Ministry of Justice Law No. 474 (May 15, 2014) concerning animal experimentation and the care of experimental animals.

## Author Contributions

EV-B-P and BK: conceptualization, methodology, validation, formal analysis, writing—original draft preparation, visualization, and supervision. EV-B-P, JK, YY, NP, and BK: investigation and data curation. EV-B-P, JK, YY, NP, HH, LA, and BK: writing—review and editing. All authors have read and agreed to the published version of the manuscript.

## Funding

This research was partly financed by the Cattle Group of the Section of Production, Nutrition and Health from the University of Copenhagen and Data Plus project fund (Strategy 2013 funds).

## Conflict of Interest

The authors declare that the research was conducted in the absence of any commercial or financial relationships that could be construed as a potential conflict of interest.

## Publisher's Note

All claims expressed in this article are solely those of the authors and do not necessarily represent those of their affiliated organizations, or those of the publisher, the editors and the reviewers. Any product that may be evaluated in this article, or claim that may be made by its manufacturer, is not guaranteed or endorsed by the publisher.
