# Supplementary material for: Goat Milk Foodomics. Dietary Supplementation of Sunflower Oil and Rapeseed Oil Modify Milk Amino Acid and Organic Acid Profiles in Dairy Goats
Source: Front Vet Sci. 2022 Mar 24;9:837229. doi: 10.3389/fvets.2022.837229 (PMC8987497; doi:10.3389/fvets.2022.837229)
Supplement: Supplementary file 1 [file Data_Sheet_1.docx]

Supplementary Material

# Supplementary Figures and Tables

Table S1. A list of identified metabolites from untargeted GC-MS foodomics of goat milk samples.

| Variables Number | Metabolite Name | Retention Time (min) | Retention Index (calculated) | Retention Index (reported) | ΔRI^#^ | Identification Level* | Metabolite Classes** | NIST match*** |
| --- | --- | --- | --- | --- | --- | --- | --- | --- |
| 1 | 3-hydroxypropionic acid | 8.47 | 1069.79 | 1057 | 12.79 | 2 | 1 | 957 |
| 2 | oxalic acid | 8.51 | 1072.5 | 1111 | -38.5 | 3 | 1 | 800 |
| 3 | UNK1 | 8.58 | 1076.98 | 0 |  | 4 | 6 | 806 |
| 4 | UNK2 | 8.56 | 1075.42 | 0 |  | 4 | 6 | 778 |
| 5 | UNK3 | 8.58 | 1076.98 | 0 |  | 3 | 6 | 779 |
| 6 | glycolic acid | 8.67 | 1082.4 | 1072 | 10.4 | 2 | 1 | 896 |
| 7 | hexanoic acid | 8.67 | 1082.5 | 1071 | 11.5 | 2 | 1 | 949 |
| 8 | UNK4 | 8.91 | 1097.4 | 0 |  | 4 | 6 | 0 |
| 9 | alanine | 9.08 | 1107.92 | 1114 | -6.08 | 2 | 2 | 871 |
| 10 | UNK5 | 9.17 | 1113.44 | 0 |  | 4 | 6 | 0 |
| 11 | UNK6 | 9.37 | 1125.94 | 0 |  | 4 | 6 | 0 |
| 12 | glycine | 9.37 | 1126.46 | 1105 | 21.46 | 2 | 2 | 912 |
| 13 | 3-hydroxybutanoic acid | 9.95 | 1162.5 | 1160 | 2.5 | 2 | 1 | 897 |
| 14 | 1-(1'-pyrrolidinyl)-2-butanone | 10.03 | 1167.71 | 1147 (estimated) |  | 2 | aminoketone | 920 |
| 15 | monomethyl phosphate | 10.19 | 1177.19 | 0 |  | 4 | phosphate | 898 |
| 16 | urea | 10.27 | 1182.19 | 1194 | -11.81 | 2 | urea | 823 |
| 17 | 2-hydroxyisovaleric acid | 10.17 | 1176.15 | 1178 | -1.85 | 3 | 1 | 786 |
| 18 | malonic acid | 10.53 | 1198.96 | 1201 | -2.04 | 2 | 1 | 852 |
| 19 | UNK7 | 10.7 | 1210.95 | 0 |  | 4 | 6 | 0 |
| 20 | UNK8 | 10.83 | 1219.88 | 0 |  | 4 | 6 | 0 |
| 21 | L-valine | 10.77 | 1215.71 | 1225.6 | -9.89 | 2 | 2 | 900 |
| 22 | dihydroxyacetone | 10.88 | 1223.81 | 1024 (estimated) |  | 3 | ketone | 883 |
| 23 | succinic acid | 12.17 | 1315.36 | 1314 | 1.36 | 2 | 1 | 919 |
| 24 | glyceric acid | 12.38 | 1330.71 | 1342 | -11.29 | 2 | 1 | 882 |
| 25 | uracil | 12.46 | 1336.43 | 1330 | 6.43 | 2 | pyrimidone | 891 |
| 26 | UNK9 | 12.52 | 1340.83 | 0 |  | 4 | 6 | 0 |
| 27 | fumaric acid | 12.65 | 1349.88 | 1345 | 4.88 | 2 | 1 | 904 |
| 28 | N,N-dimethyl-2-phenylmethoxyethanamine | 12.72 | 1355 | 1316 (estimated) |  | 3 | amine der. | 821 |
| 29 | serine | 12.79 | 1360.12 | 1369.5 | -9.38 | 2 | 2 | 892 |
| 30 | UNK10 | 13.05 | 1378.57 | 0 |  | 4 | 6 | 0 |
| 31 | UNK11 | 13.06 | 1378.93 | 0 |  | 4 | 6 | 0 |
| 32 | L-threonine | 13.13 | 1384.29 | 1367.4 | 16.89 | 2 | 2 | 882 |
| 33 | UNK12 | 13.2 | 1388.93 | 0 |  | 4 | 6 | 0 |
| 34 | UNK13 | 13.31 | 1397.38 | 0 |  | 4 | 6 | 0 |
| 35 | dihydroxypropanal O-methyloxime | 13.62 | 1421.82 | 1215 (estimated) |  | 3 | oxime | 777 |
| 36 | UNK14 | 13.86 | 1440.96 | 0 |  | 4 | 6 | 0 |
| 37 | capric acid | 14.06 | 1456.89 | 1455 | 1.89 | 2 | 3 | 894 |
| 38 | UNK15 | 14.13 | 1462.78 | 0 |  | 4 | 6 | 0 |
| 39 | 2,3-dihydroxypropyl 2-methylpropanoate der. | 14.21 | 1468.94 | 1324 (estimated) |  | 3 | ester | 870 |
| 40 | UNK16 | 14.19 | 1467.6 | 0 |  | 4 | 6 | 0 |
| 41 | aminomalonic acid | 14.19 | 1467.47 | 1413 (estimated) |  | 3 | 2 | 815 |
| 42 | UNK17 | 14.32 | 1477.64 | 0 |  | 4 | 6 | 0 |
| 43 | malic acid | 14.44 | 1487.15 | 1390 (estimated) |  | 3 | 1 | 904 |
| 44 | UNK18 | 14.48 | 1491.03 | 0 |  | 4 | 6 | 0 |
| 45 | UNK19 | 14.68 | 1507.1 | 0 |  | 4 | 6 | 0 |
| 46 | UNK20 | 14.74 | 1511.78 | 0 |  | 4 | 6 | 0 |
| 47 | L-pyroglutamic acid | 14.81 | 1517.54 | 1520.7 | -3.16 | 3 | 2 | 767 |
| 48 | L-pyroglutamic acid | 14.82 | 1518.21 | 1520.7 | -2.49 | 2 | 2 | 927 |
| 49 | 3-hydroxyproline | 14.89 | 1523.43 | 1504 | 19.43 | 2 | 2 | 892 |
| 50 | pyroglutamic acid | 14.97 | 1530.25 | 1511 | 19.25 | 3 | 2 | 798 |
| 51 | UNK21 | 15.1 | 1540.43 | 0 |  | 4 | 6 | 0 |
| 52 | L-threonic acid | 15.31 | 1557.16 | 1523.2 | 33.96 | 3 | 4 | 847 |
| 53 | UNK22 | 15.37 | 1561.98 | 0 |  | 4 | 6 | 0 |
| 54 | serine | 15.48 | 1571.08 | 1580 | -8.92 | 2 | 2 | 865 |
| 55 | UNK23 | 15.54 | 1576.04 | 0 |  | 4 | 6 | 0 |
| 56 | 2-propionylbenzoic acid | 15.68 | 1587.42 | 1598 | -10.58 | 2 | 1 | 862 |
| 57 | UNK24 | 15.79 | 1596.12 | 0 |  | 3 | 6 | 782 |
| 58 | α-ketoglutaric acid | 15.86 | 1602.11 | 1622 | -19.89 | 2 | 1 | 860 |
| 59 | UNK25 | 15.9 | 1604.98 | 0 |  | 4 | 6 | 0 |
| 60 | L-glutamic acid | 16.02 | 1615.99 | 1629.4 | -13.41 | 2 | 2 | 862 |
| 61 | L-phenylalanine | 16.06 | 1620.21 | 1710 (estimated) |  | 3 | 2 | 815 |
| 62 | UNK26 | 16.37 | 1647.51 | 0 |  | 4 | 6 | 0 |
| 63 | D-xylose | 16.57 | 1665.76 | 1692 (estimated) |  | 2 | 4 | 855 |
| 64 | UNK27 | 16.64 | 1672.55 | 0 |  | 4 | 6 | 0 |
| 65 | α-ketoglutaric acid | 16.67 | 1674.81 | 1386 (estimated) |  | 3 | 1 | 806 |
| 66 | UNK28 | 16.87 | 1692.76 | 0 |  | 4 | 6 | 0 |
| 67 | β-L-(-)-fucopyranose | 17.02 | 1706.49 | 1697.4 | 9.09 | 2 | 4 | 829 |
| 68 | ribitol | 16.99 | 1704.22 | 1727.1 | -22.88 | 2 | 5 | 845 |
| 69 | ribitol | 17.15 | 1718.25 | 1727.1 | -8.85 | 2 | 5 | 918 |
| 70 | ribitol | 17.22 | 1724.59 | 1727.1 | -2.51 | 2 | 5 | 917 |
| 71 | orotic acid | 17.4 | 1740.87 | 1740 | 0.87 | 2 | 1 | 897 |
| 72 | UNK29 | 17.45 | 1745.4 | 0 |  | 4 | 6 | 0 |
| 73 | L-(-)-sorbofuranose | 17.66 | 1764.4 | 1778.8 | -14.4 | 3 | 4 | 773 |
| 74 | glycerol 1-phosphate | 17.62 | 1760.94 | 1744 | 16.94 | 2 | phosphate | 871 |
| 75 | α-D-mannopyranose | 17.89 | 1785.67 | 1793.6 | -7.93 | 2 | 4 | 832 |
| 76 | UNK30 | 18.11 | 1805.37 | 0 |  | 4 | 6 | 0 |
| 77 | α-D-allopyranose | 18.08 | 1802.44 | 1814.3 | -11.86 | 2 | 4 | 852 |
| 78 | citric acid | 18.25 | 1819.35 | 1839 | -19.65 | 2 | 1 | 883 |
| 79 | UNK31 | 18.43 | 1837.07 | 0 |  | 4 | 6 | 0 |
| 80 | hippuric acid | 18.49 | 1842.6 | 1805 | 37.6 | 3 | 1 | 861 |
| 81 | D-(+)-galactopyranose | 18.62 | 1855.61 | 1846.7 | 8.91 | 2 | 4 | 880 |
| 82 | β-D-(+)-mannopyranose | 18.97 | 1889.92 | 1890.3 | -0.38 | 2 | 4 | 889 |
| 83 | β-D-(+)-talopyranose | 19.07 | 1899.84 | 1895.9 | 3.94 | 2 | 4 | 885 |
| 84 | UNK32 | 19.2 | 1912.03 | 0 |  | 4 | 6 | 0 |
| 85 | UNK33 | 19.63 | 1953.98 | 0 |  | 4 | 6 | 0 |
| 86 | D-gluconic acid | 19.71 | 1962.28 | 2009.7 | -47.42 | 3 | 4 | 816 |
| 87 | D-glucono-1,4-lactone | 19.7 | 1960.98 | 1932 | 28.98 | 2 | 4 | 804 |
| 88 | β-D-glucopyranose | 19.84 | 1974.63 | 2009.5 | -34.87 | 3 | 4 | 894 |
| 89 | UNK34 | 20.03 | 1992.85 | 0 |  | 4 | 6 | 0 |
| 90 | UNK35 | 20.32 | 2018.19 | 0 |  | 4 | 6 | 0 |
| 91 | scyllo-inositol | 20.35 | 2020.69 | 2194 (estimated) |  | 3 | 5 | 844 |
| 92 | palmitic acid | 20.49 | 2032.36 | 2039.1 | -6.74 | 2 | 3 | 891 |
| 93 | myo-inositol | 21.02 | 2076.25 | 2153 | -76.75 | 3 | 5 | 942 |
| 94 | myo-inositol | 21.21 | 2092.78 | 2153 | -60.22 | 3 | 5 | 963 |
| 95 | UNK36 | 21.52 | 2118.33 | 0 |  | 4 | 6 | 0 |
| 96 | 2-pentadecyl-1,3-dioxolane | 21.92 | 2151.39 | 2030 (estimated) |  | 3 | 3 | 780 |
| 97 | UNK37 | 22.16 | 2171.25 | 0 |  | 3 | 6 | 761 |

# Difference between calculated and reported RI.

* Level 2 when the peaks are identified based on mass spectral (EI-MS) similarity of ≥ 80% and RI match of ±30; Level 3 when the peaks are identified at metabolite class level only based on mass spectral match of ≥ 65%. Level 4 unidentified peaks.

** 1=Organic acids, 2=Amino Acids, 3=Fatty Acids, 4=Sugars, 5=Sugar alcohols, 6=UNK.

*** Mass spectral (EI-MS) match of identified metabolites using NIST11 metabolite library.

Table 2S. A list of identified metabolites from NMR foodomics of goat milk samples.

| Variables Numbers | Metabolite name | Class^#^ | End ppm | Start ppm | Multiplicity* | Type of protons | Metabolite class** |
| --- | --- | --- | --- | --- | --- | --- | --- |
| 1 | butanoic acid 1 | SS | 0.91 | 0.89 | t | CH3 | 3 |
| 2 | valine1 | SS | 1.01 | 0.99 | d | CH3 | 2 |
| 3 | isoleucine1 | SS | 1.03 | 1.01 | d | CH3 | 2 |
| 4 | valine2 | SS | 1.06 | 1.04 | d | CH3 | 2 |
| 5 | ethanol | SS | 1.19 | 1.16 | d(t) | CH3 | 7 |
| 6 | isobutyric acid | SS | 1.24 | 1.22 | d | CH3 | 3 |
| 7 | capric acid | SS | 1.31 | 1.27 | br,s | CH2 | 3 |
| 8 | lactic acid | SS | 1.35 | 1.32 | d | CH3 | 1 |
| 9 | isoleucine2 | SS | 1.47 | 1.43 | m | γ-CH2 | 2 |
| 10 | alanine | SS | 1.51 | 1.47 | d | CH3 | 2 |
| 11 | butanoic acid 2 | SS | 1.58 | 1.53 | m | CH2 | 3 |
| 12 | ornithine | SS | 1.78 | 1.72 | m | CH2 | 2 |
| 13 | ornithine2 | SS | 1.92 | 1.85 | m | CH2 | 2 |
| 14 | acetic acid | SS | 1.93 | 1.92 | s | CH3 | 3 |
| 15 | methionine | SS | 2.19 | 2.15 | m | (part of) CH3, CH2 | 2 |
| 16 | acetone | SS | 2.24 | 2.24 | s | CH3 | 7 |
| 17 | lipid | SS | 2.34 | 2.24 | br,s | -CH2-C = O | 3 |
| 18 | glutamic acid | SS | 2.38 | 2.34 | m | CH2 | 2 |
| 19 | oxaloacetic acid | SS | 2.39 | 2.38 | s | CH2 | 1 |
| 20 | succinic acid | SS | 2.41 | 2.40 | s | CH2 | 1 |
| 21 | glutamine | SS | 2.49 | 2.43 | m | CH2 | 2 |
| 22 | citric acid 1 | SS | 2.56 | 2.52 | d | CH2 | 1 |
| 23 | methylamine | SS | 2.62 | 2.61 | s | CH3 | 6 |
| 24 | citric acid 2 | SS | 2.67 | 2.65 | s(d) | ½ CH2 | 1 |
| 25 | citric acid 3 | SS | 2.70 | 2.68 | s(d) | ½ CH2 | 1 |
| 26 | dimethylamine | SS | 2.73 | 2.72 | s | CH3 | 6 |
| 27 | 2-oxoglutaric acid | SS | 3.02 | 2.99 | t | CH2 | 1 |
| 28 | creatine | SS | 3.05 | 3.04 | s | CH3 | 2 |
| 29 | creatinine | SS | 3.06 | 3.05 | s | CH3 | 2 |
| 30 | malonic acid | SS | 3.12 | 3.11 | s | CH2 | 1 |
| 31 | choline1 | SS | 3.20 | 3.19 | s | CH3 | 6 |
| 32 | glycerophosphocholine | SS | 3.21 | 3.20 | s | CH3 | 6 |
| 33 | carnitine | SS | 3.23 | 3.21 | s | CH3 | 6 |
| 34 | acetylcholine | SS | 3.24 | 3.23 | s | CH3 | 6 |
| 35 | taurine1 | SS | 3.26 | 3.26 | s(t) | CH2 | 1 |
| 36 | taurine2 | SS | 3.28 | 3.27 | s(t) | CH2 | 1 |
| 37 | lactose1 | SS | 3.34 | 3.28 | m | CH | 4 |
| 38 | methanol | SS | 3.35 | 3.34 | s | CH3 | 7 |
| 39 | methylguanidine | SS | 3.37 | 3.36 | s | =N-CH3 | 6 |
| 40 | taurine3 | SS | 3.43 | 3.41 | t | CH2 | 1 |
| 41 | uridine diphosphate glucose | SS | 3.53 | 3.45 | tt | N/A | 5 |
| 42 | lactose2 | SS | 3.60 | 3.54 | m | CH | 4 |
| 43 | lactose3 | SS | 3.63 | 3.60 | m | CH | 4 |
| 44 | lactose4 | SS | 3.71 | 3.64 | m | CH | 4 |
| 45 | lactose5 | SS | 3.78 | 3.72 | m | CH | 4 |
| 46 | lactose6 | SS | 3.85 | 3.78 | m | CH | 4 |
| 47 | lactose7 | SS | 3.94 | 3.85 | m | CH | 4 |
| 48 | lactose8 | SS | 4.00 | 3.94 | m | CH | 4 |
| 49 | bin6 | BINS | 4.07 | 4.02 | m | CH2 | N/A |
| 50 | malic acid | SS | 4.30 | 4.28 | dd | CH | 1 |
| 51 | lactose9 | SS | 4.49 | 4.44 | dd | CH | 4 |
| 52 | β-galactose | SS | 4.56 | 4.53 | d | CH | 4 |
| 53 | β-glucose | SS | 4.61 | 4.59 | d | CH | 4 |
| 54 | mannose | SS | 5.12 | 5.10 | d | CH2 | 4 |
| 55 | glucose-1-phosphate | SS | 5.54 | 5.51 | m | CH | 4 |
| 56 | urea | SS | 5.71 | 5.69 | br,s | NH2 | 6 |
| 57 | uridine | SS | 5.93 | 5.91 | m | CH2 | 5 |
| 58 | uridine diphosphate-N-acetylglucosamine1 | SS | 6.00 | 5.96 | m | CH | 5 |
| 59 | orotic acid | SS | 6.20 | 6.19 | s | -C=CH | 1 |
| 60 | fumaric acid | SS | 6.53 | 6.52 | s | HC=CH | 1 |
| 61 | tyrosine1 | SS | 6.93 | 6.90 | d | (CH)2 | 2 |
| 62 | tyrosine2 | SS | 7.22 | 7.19 | d | (CH)2 | 2 |
| 63 | phenylalanine | SS | 7.40 | 7.26 | m | (=CH)5 | 2 |
| 64 | hippuric acid 1 | SS | 7.59 | 7.54 | t | CH2, CH | 1 |
| 65 | hippuric acid 2 | SS | 7.67 | 7.63 | m | CH | 1 |
| 66 | hippuric acid 3 | SS | 7.88 | 7.83 | m | CH2, CH | 1 |
| 67 | uridine diphosphate-N-acetylglucosamine2 | SS | 7.95 | 7.92 | tt | CH | 5 |
| 68 | adenine | SS | 8.10 | 8.07 | d | CH | 5 |
| 69 | formic acid | SS | 8.50 | 8.49 | s | CH | 3 |
| 70 | UNK1 | SUS | 2.09 | 2.08 | s | N/A | N/A |
| 71 | UNK2 | SUS | 2.68 | 2.67 | s | N/A | N/A |
| 72 | UNK3 | SUS | 2.72 | 2.71 | s | N/A | N/A |
| 73 | UNK4 | SUS | 3.17 | 3.16 | s | N/A | N/A |
| 74 | UNK5 | SUS | 3.36 | 3.35 | s | N/A | N/A |
| 75 | UNK6 | SUS | 3.38 | 3.37 | s | N/A | N/A |
| 76 | UNK7 | SUS | 4.22 | 4.19 | m | N/A | N/A |
| 77 | UNK8 | SUS | 4.34 | 4.32 | dd | N/A | N/A |
| 78 | UNK9 | SUS | 4.40 | 4.34 | m | N/A | N/A |
| 79 | UNK10 | SUS | 4.83 | 4.80 | d | N/A | N/A |
| 80 | UNK11 | SUS | 4.94 | 4.90 | t | N/A | N/A |
| 81 | UNK12 | SUS | 5.40 | 5.38 | t | N/A | N/A |
| 82 | UNK13 | SUS | 5.57 | 5.54 | q | N/A | N/A |
| 83 | UNK14 | SUS | 5.62 | 5.59 | q | N/A | N/A |
| 84 | UNK15 | SUS | 5.63 | 5.62 | d | N/A | N/A |
| 85 | UNK16 | SUS | 5.66 | 5.63 | q | N/A | N/A |
| 86 | UNK17 | SUS | 5.83 | 5.80 | d | N/A | N/A |
| 87 | UNK18 | SUS | 5.96 | 5.93 | dd | N/A | N/A |
| 88 | UNK19 | SUS | 6.11 | 6.09 | d | N/A | N/A |
| 89 | UNK20 | SUS | 8.12 | 8.10 | s | N/A | N/A |
| 90 | UNK21 | SUS | 8.26 | 8.25 | s | N/A | N/A |
| 91 | UNK22 | SUS | 8.29 | 8.28 | s | N/A | N/A |
| 92 | UNK23 | SUS | 8.31 | 8.30 | d | N/A | N/A |
| 93 | UNK24 | SUS | 8.35 | 8.34 | s | N/A | N/A |
| 94 | UNK25 | SUS | 8.54 | 8.53 | s | N/A | N/A |
| 95 | UNK26 | SUS | 8.56 | 8.55 | s | N/A | N/A |
| 96 | UNK27 | SUS | 8.60 | 8.58 | s | N/A | N/A |
| 97 | bin-caprylic acid,lipid,Caproic acid (CH3) | BINS | 0.89 | 0.85 | N/A | CH3 | N/A |
| 98 | bin-Isoleucine (δ-CH3), Leucine (CH3) | BINS | 0.94 | 0.92 | N/A | CH3 | N/A |
| 99 | bin-2-Aminobutyric acid (γ-CH3),pantothenic acid | BINS | 0.98 | 0.94 | N/A | N/A | N/A |
| 100 | bin-β-Fucose,3-Hydroxybutyric acid (CH3) | BINS | 1.22 | 1.19 | N/A | CH3 | N/A |
| 101 | bin-β-Fucose(CH3) | BINS | 1.26 | 1.24 | N/A | CH3 | N/A |
| 102 | bin1 | BINS | 1.43 | 1.39 | N/A | N/A | N/A |
| 103 | bin-N-acetyl-D-glucosamine(CH2) | BINS | 2.06 | 2.04 | N/A | CH3 | N/A |
| 104 | bin-glutamic acid,N-acetylsugar B(CH3) | BINS | 2.07 | 2.06 | N/A | CH3 | N/A |
| 105 | bin-glutamic acid,N-acetylsugar C(CH3) | BINS | 2.08 | 2.07 | N/A | CH3 | N/A |
| 106 | bin-Glutamic acid (β-CH2), Glutamine (β-CH2) | BINS | 2.15 | 2.10 | N/A | β-CH2 | N/A |
| 107 | bin-Methionine,acetylcholine | BINS | 2.15 | 2.15 | N/A | N/A | N/A |
| 108 | bin-taurine,betaine | BINS | 3.27 | 3.26 | N/A | N/A | N/A |
| 109 | bin2 | BINS | 3.44 | 3.43 | N/A | N/A | N/A |
| 110 | bin-galactose,creatinie | BINS | 4.09 | 4.07 | N/A | N/A | N/A |
| 111 | bin-lactic acid (CH) | BINS | 4.15 | 4.10 | N/A | CH | N/A |
| 112 | bin-3-hydroxybutyric acid,phosphocholine | BINS | 4.19 | 4.15 | N/A | N/A | N/A |
| 113 | bin3 | BINS | 4.25 | 4.23 | N/A | N/A | N/A |
| 114 | bin4 | BINS | 4.28 | 4.25 | N/A | N/A | N/A |
| 115 | bin-lactose,galactose,β-glucose | BINS | 5.27 | 5.22 | N/A | N/A | N/A |
| 116 | bin5 | BINS | 6.16 | 6.13 | N/A | N/A | N/A |

# SS are SigMa based identified spectral intervals corresponding to Signature Signals (SS); SUS are identified spectral intervals corresponding to Signals of Unknown Spin systems (SUS) using SigMa’s Chemical Shift Library developed in-house; BINS are bins of complex unresolved regions (BINS).

* br, s= broad singlet; s=singlet, d=doublet, t=triplet, dd=doublet of doublet, tt=triplet of triplet, m=multiplet, s(d)=singlet of doublet, s(t)=singlet of triplet, d(t)=doublet of triplet

**1 = Organic acids, 2 = Amino Acids, 3 = Fatty Acids, 4 = Sugars, 5 = Purine & Pyrimidine der., 6 = Amine & Quaternary ammonium der., 7 = Others.


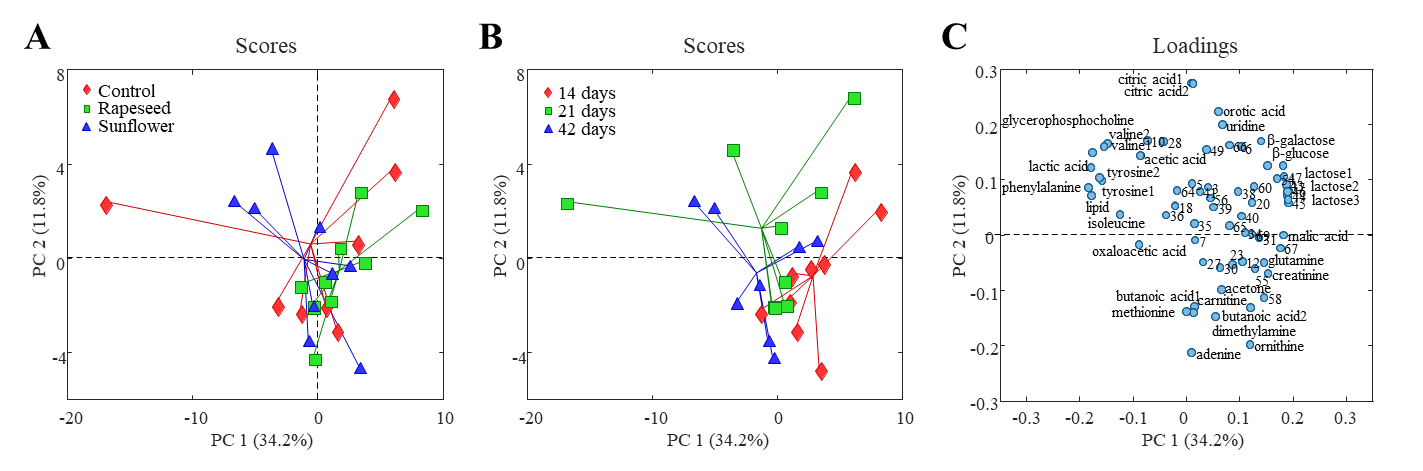


Figure S1. Score and loading plots of the PCA model developed on goat milk NMR foodomics data. (A) Principal component 1 (PC1) versus PC2 scores plot and milk samples are color coded according to the dietary treatment and in panel (B) milk samples are colored according to the experimental period. (C) PC1 versus PC2 loadings plot from the corresponding PCA model (variables are numbered as in Table S2).


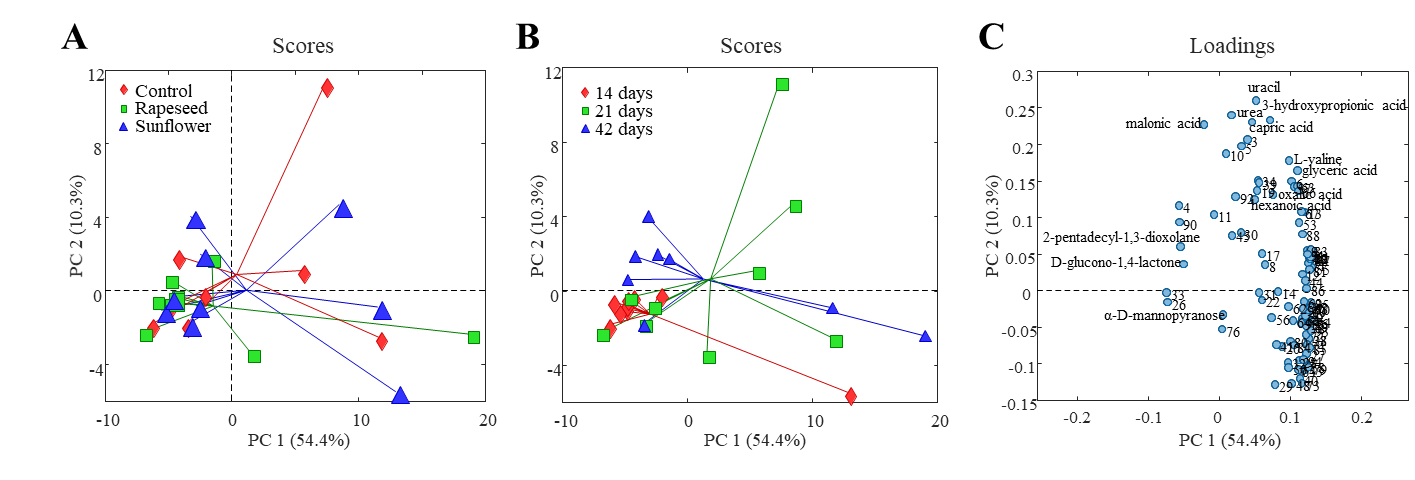


Figure S2. Score and loading plots of the PCA model developed on goat milk GC-MS foodomics data. (A) Principal component 1 (PC1) versus PC2 scores plot and milk samples are color coded according to the dietary treatment and in panel (B) milk samples are colored according to the experimental period. (C) PC1 versus PC2 loadings plot from the corresponding PCA model (variables are numbered as in Table S1).

**
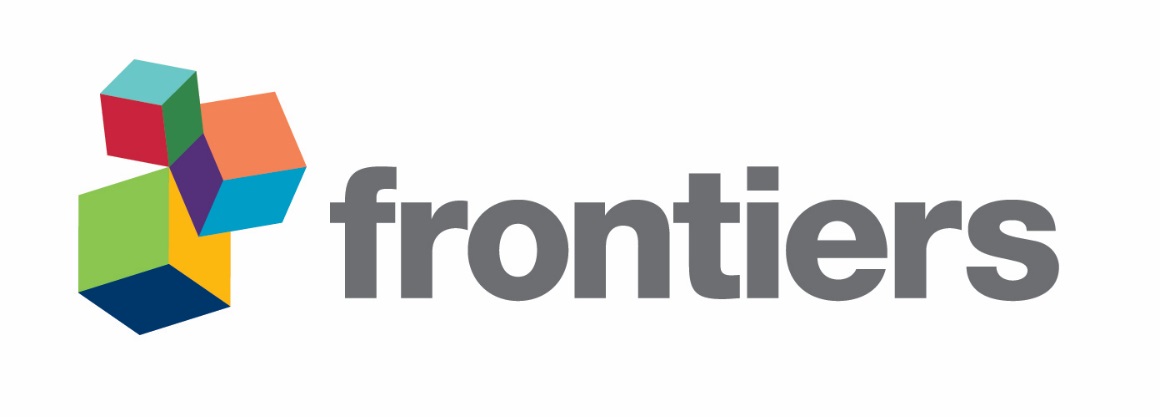
**
